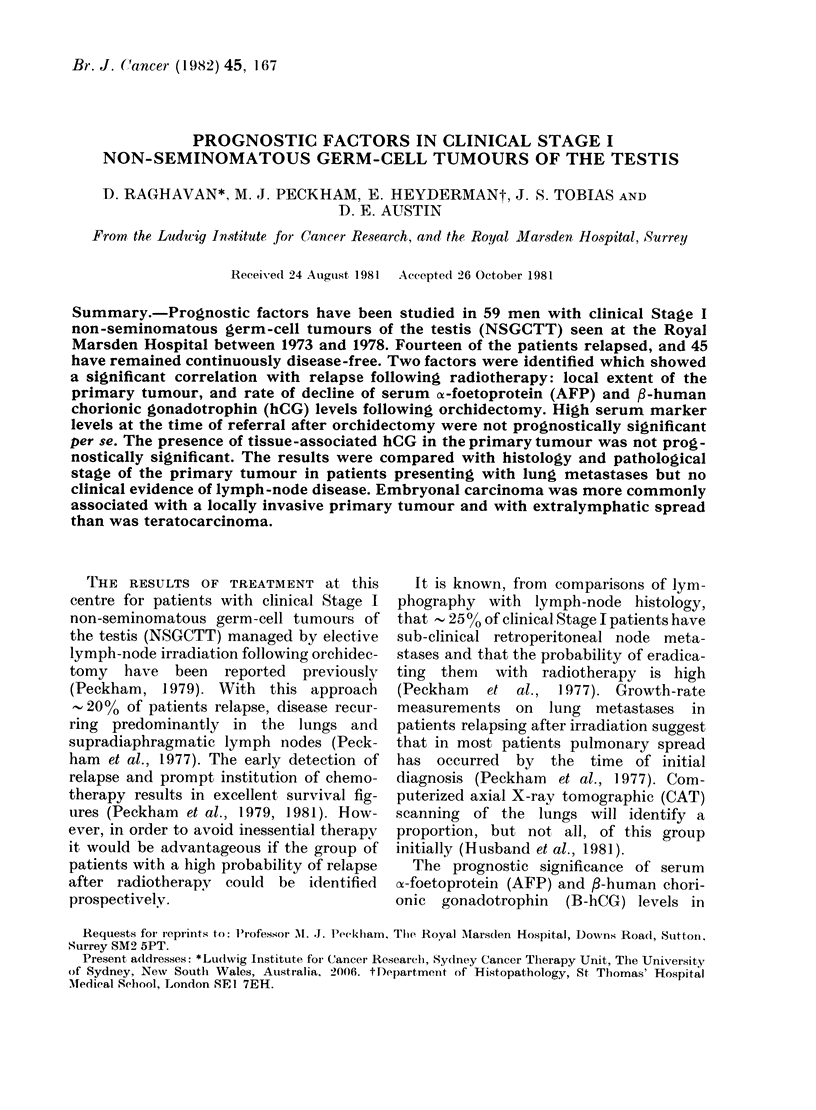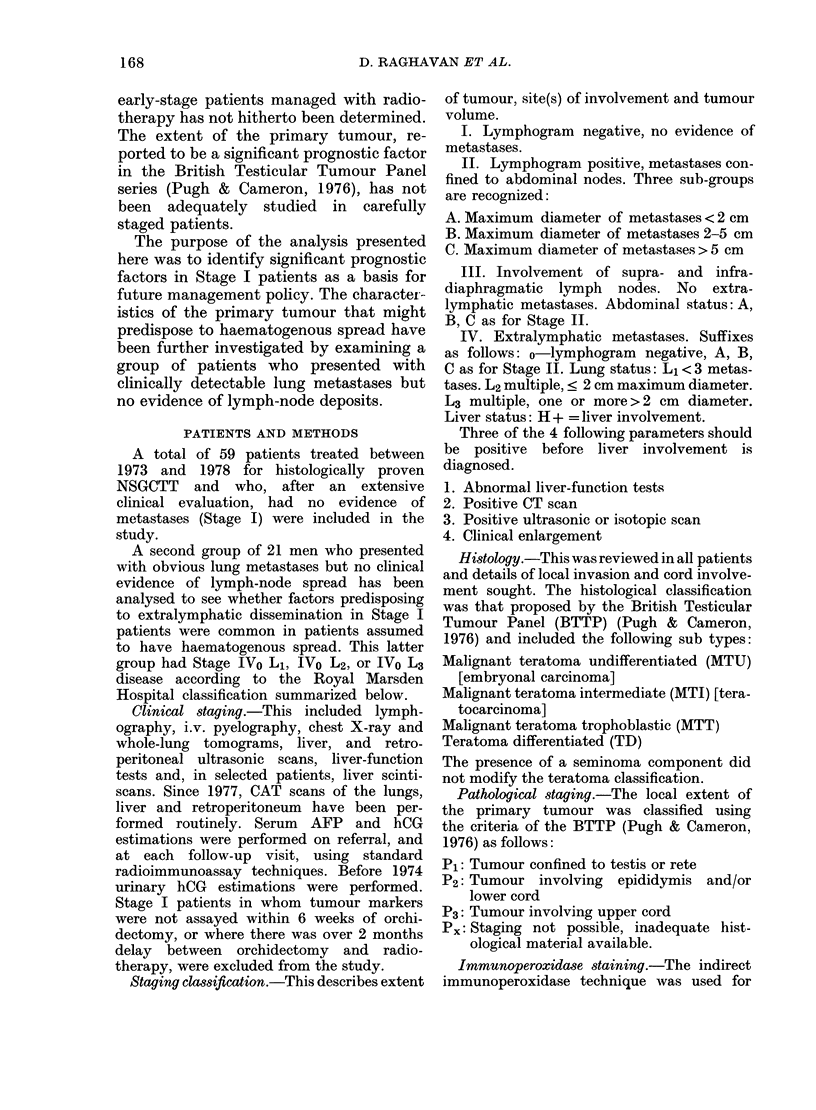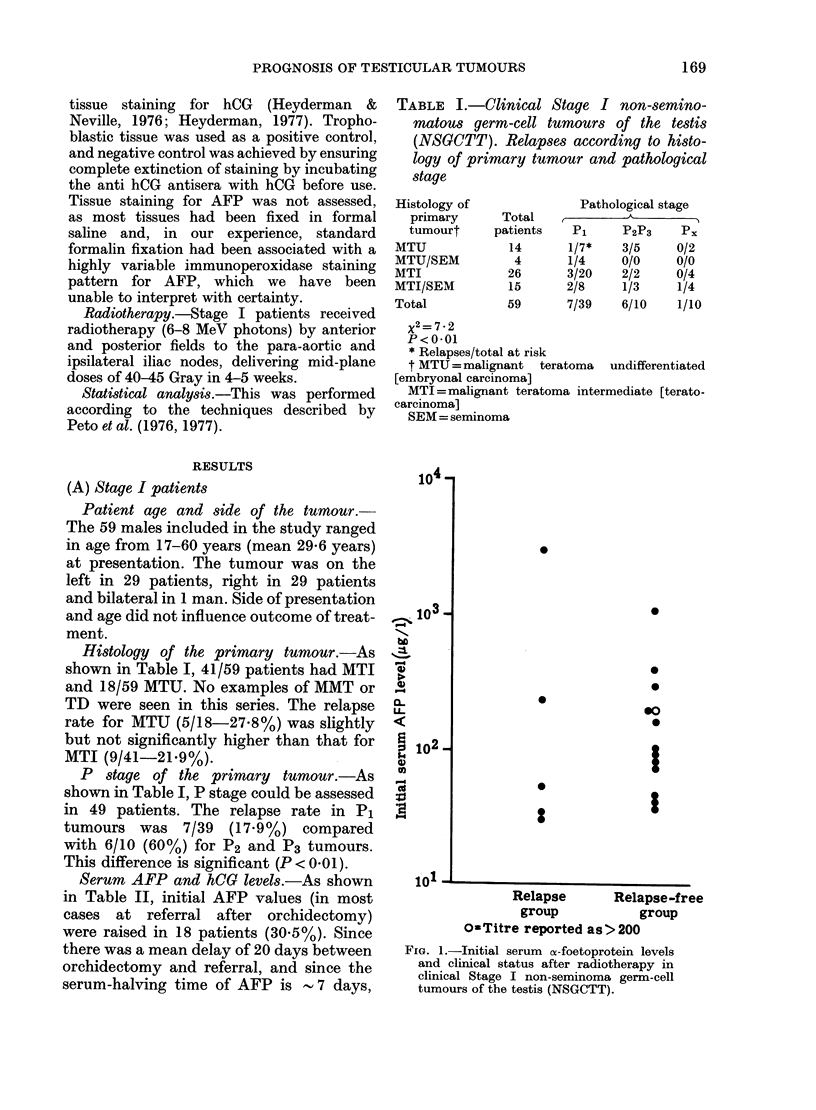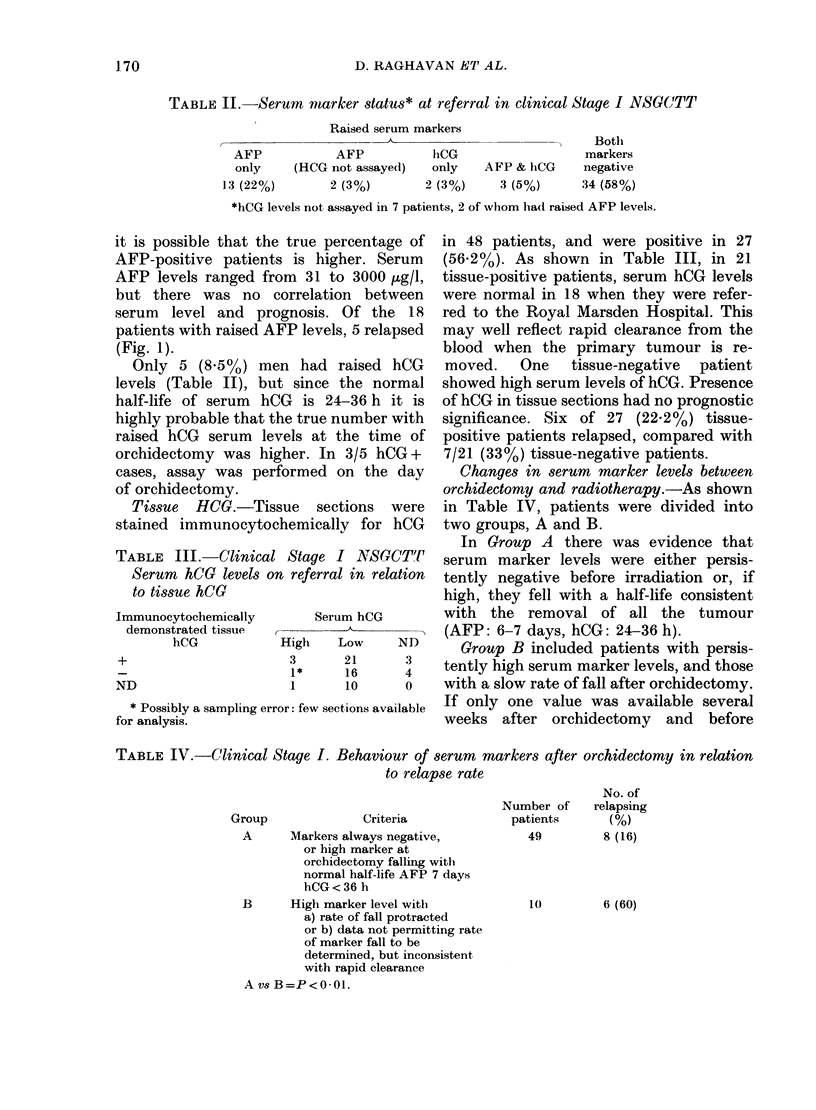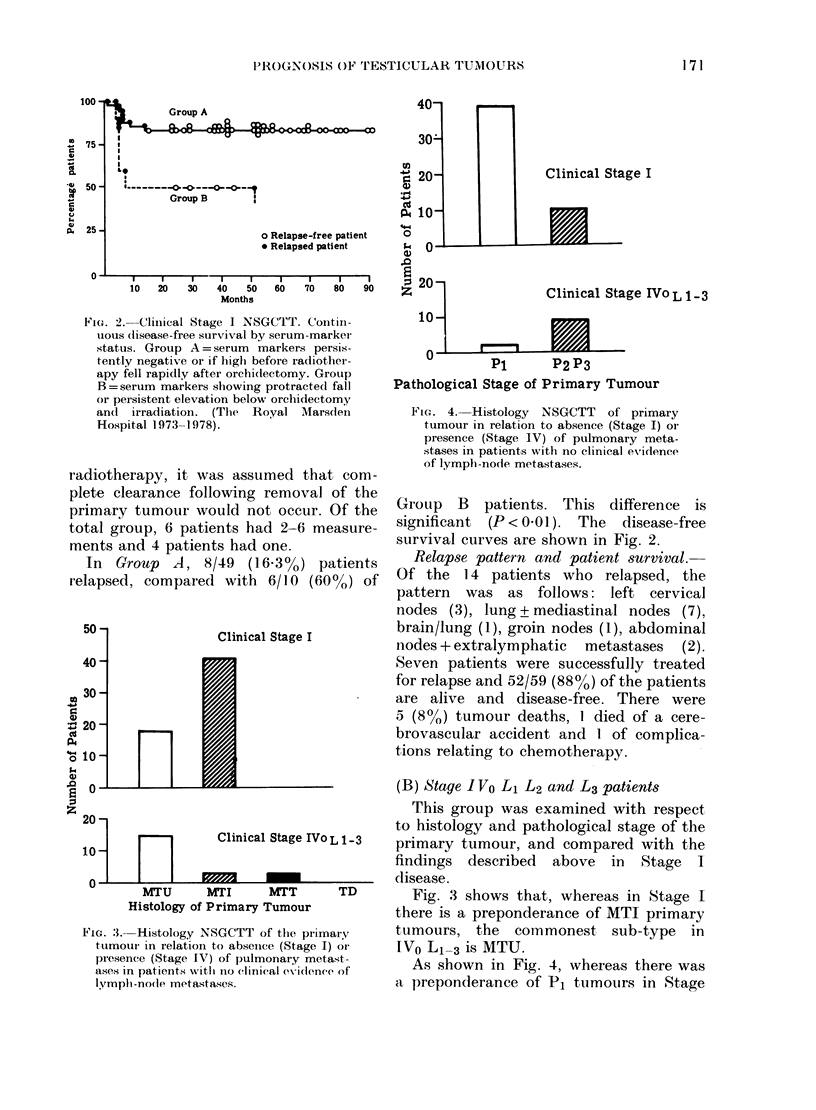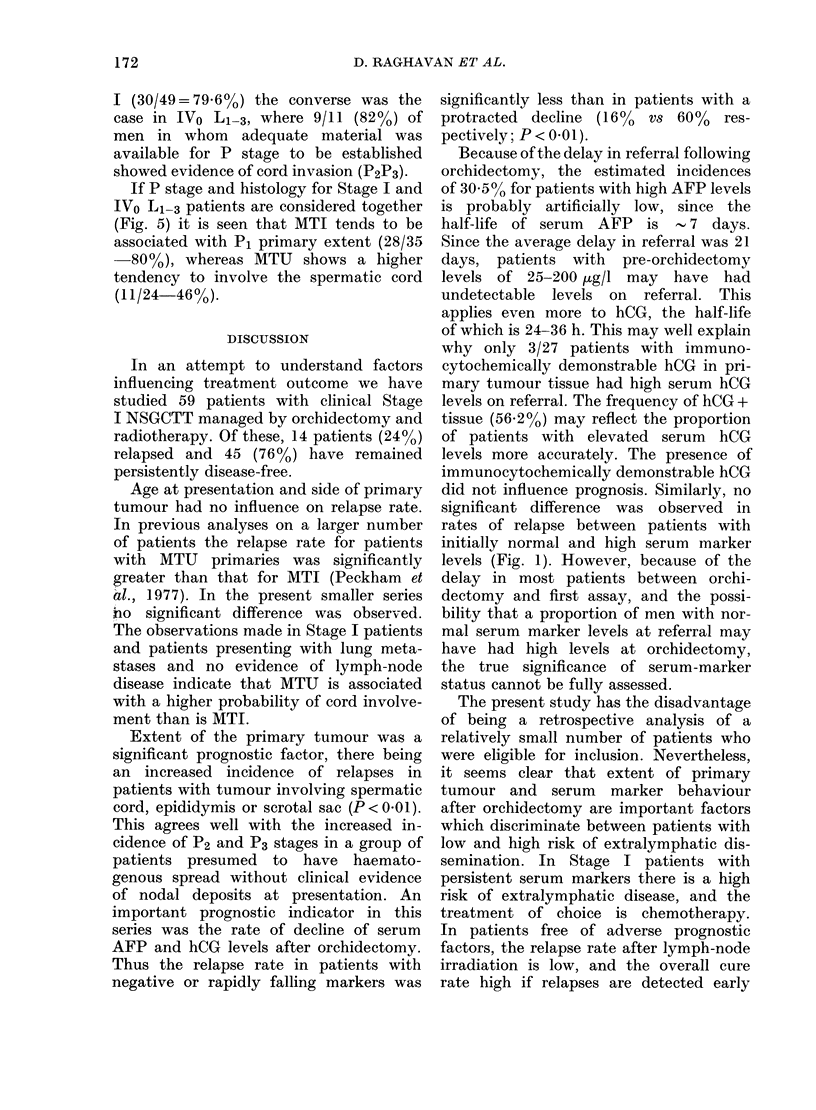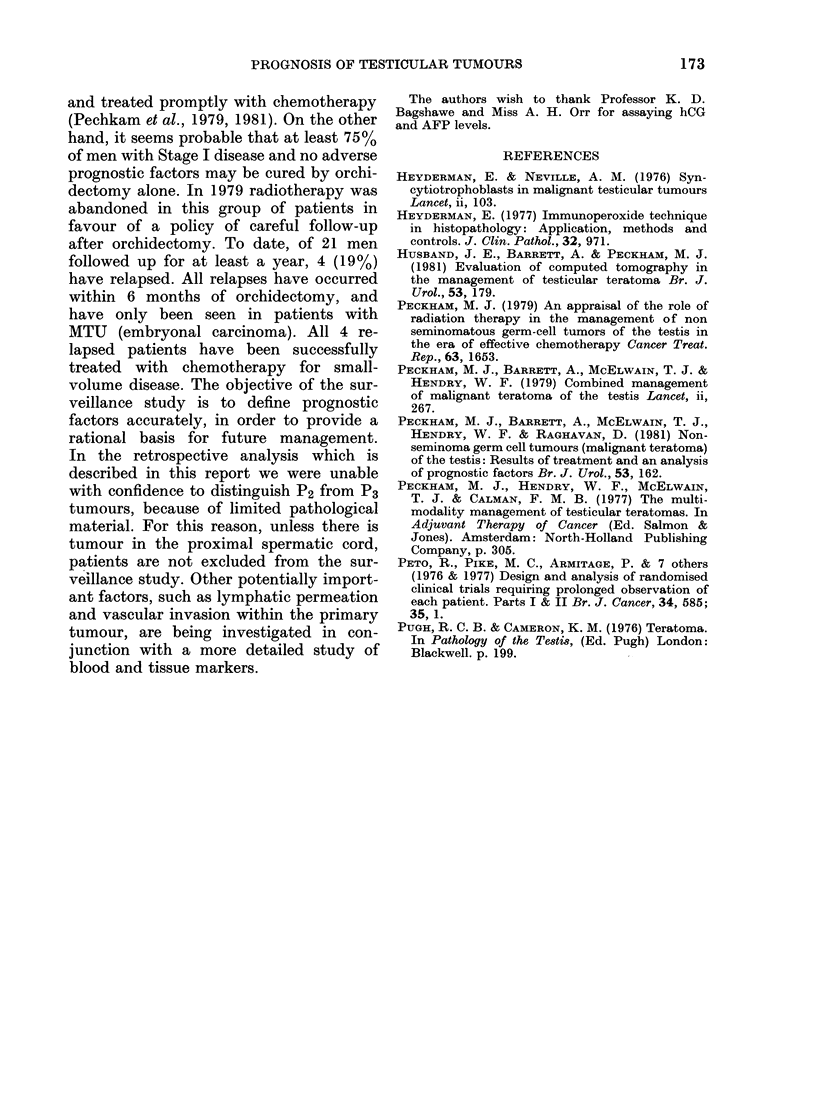# Prognostic factors in clinical stage I non-seminomatous germ-cell tumours of the testis.

**DOI:** 10.1038/bjc.1982.29

**Published:** 1982-02

**Authors:** D. Raghavan, M. J. Peckham, E. Heyderman, J. S. Tobias, D. E. Austin

## Abstract

Prognostic factors have been studied in 59 men with clinical Stage I non-seminomatous germ-cell tumours of the testis (NSGCTT) seen at the Royal Marsden Hospital between 1973 and 1978. Fourteen of the patients relapsed, and 45 have remained continuously disease-free. Two factors were identified which showed a significant correlation with relapse following radiotherapy: local extent of the primary tumour, and rate of decline of serum alpha-foetoprotein (AFP) and beta-human chorionic gonadotrophin (hCG) levels following orchidectomy. High serum marker levels at the time of referral after orchidectomy were not prognostically significant per se. The presence of tissue-associated hCG in the primary tumour was not prognostically significant. The results were compared with histology and pathological stage of the primary tumour in patients presenting with lung metastases but no clinical evidence of lymph-node disease. Embryonal carcinoma was more commonly associated with a locally invasive primary tumour and with extralymphatic spread than was teratocarcinoma.


*Br. J. Cancer* (1982) **45**, 167

### PROGNOSTIC FACTORS IN CLINICAL STAGE I NON-SEMINOMATOUS GERM-CELL TUMOURS OF THE TESTIS

D. RAGHAVAN*, M. J. PECKHAM, E. HEYDERMAN†, J. S. TOBIAS AND D. E. AUSTIN

*From the Ludwig Institute for Cancer Research, and the Royal Marsden Hospital, Surrey*

Received 24 August 1981 Accepted 26 October 1981

**Summary.—Prognostic factors have been studied in 59 men with clinical Stage I non-seminomatous germ-cell tumours of the testis (NSGCTT) seen at the Royal Marsden Hospital between 1973 and 1978. Fourteen of the patients relapsed, and 45 have remained continuously disease-free. Two factors were identified which showed a significant correlation with relapse following radiotherapy: local extent of the primary tumour, and rate of decline of serum α-foetoprotein (AFP) and β-human chorionic gonadotrophin (hCG) levels following orchidectomy. High serum marker levels at the time of referral after orchidectomy were not prognostically significant *per se*. The presence of tissue-associated hCG in the primary tumour was not prognostically significant. The results were compared with histology and pathological stage of the primary tumour in patients presenting with lung metastases but no clinical evidence of lymph-node disease. Embryonal carcinoma was more commonly associated with a locally invasive primary tumour and with extralymphatic spread than was teratocarcinoma.**

THE RESULTS OF TREATMENT at this centre for patients with clinical Stage I non-seminomatous germ-cell tumours of the testis (NSGCTT) managed by elective lymph-node irradiation following orchidectomy have been reported previously (Peckham, 1979). With this approach ~20% of patients relapse, disease recurring predominantly in the lungs and supradiaphragmatic lymph nodes (Peckham *et al.*, 1977). The early detection of relapse and prompt institution of chemotherapy results in excellent survival figures (Peckham *et al.*, 1979, 1981). However, in order to avoid inessential therapy it would be advantageous if the group of patients with a high probability of relapse after radiotherapy could be identified prospectively.

It is known, from comparisons of lymphography with lymph-node histology, that ~25% of clinical Stage I patients have sub-clinical retroperitoneal node metastases and that the probability of eradicating them with radiotherapy is high (Peckham *et al.*, 1977). Growth-rate measurements on lung metastases in patients relapsing after irradiation suggest that in most patients pulmonary spread has occurred by the time of initial diagnosis (Peckham *et al.*, 1977). Computerized axial X-ray tomographic (CAT) scanning of the lungs will identify a proportion, but not all, of this group initially (Husband *et al.*, 1981).

The prognostic significance of serum α-foetoprotein (AFP) and β-human chorionic gonadotrophin (B-hCG) levels in

Requests for reprints to: Professor M. J. Peckham, The Royal Marsden Hospital, Downs Road, Sutton, Surrey SM2 5PT.

Present addresses: *Ludwig Institute for Cancer Research, Sydney Cancer Therapy Unit, The University of Sydney, New South Wales, Australia, 2006. †Department of Histopathology, St Thomas' Hospital Medical School, London SE1 7EH.

early-stage patients managed with radiotherapy has not hitherto been determined. The extent of the primary tumour, reported to be a significant prognostic factor in the British Testicular Tumour Panel series (Pugh & Cameron, 1976), has not been adequately studied in carefully staged patients.

The purpose of the analysis presented here was to identify significant prognostic factors in Stage I patients as a basis for future management policy. The characteristics of the primary tumour that might predispose to haematogenous spread have been further investigated by examining a group of patients who presented with clinically detectable lung metastases but no evidence of lymph-node deposits.

### PATIENTS AND METHODS

A total of 59 patients treated between 1973 and 1978 for histologically proven NSGCTT and who, after an extensive clinical evaluation, had no evidence of metastases (Stage I) were included in the study.

A second group of 21 men who presented with obvious lung metastases but no clinical evidence of lymph-node spread has been analysed to see whether factors predisposing to extralymphatic dissemination in Stage I patients were common in patients assumed to have haematogenous spread. This latter group had Stage $IV_0$ $L_1$, $IV_0$ $L_2$, or $IV_0$ $L_3$ disease according to the Royal Marsden Hospital classification summarized below.

*Clinical staging.*—This included lymphography, i.v. pyelography, chest X-ray and whole-lung tomograms, liver, and retroperitoneal ultrasonic scans, liver-function tests and, in selected patients, liver scintiscans. Since 1977, CAT scans of the lungs, liver and retroperitoneum have been performed routinely. Serum AFP and hCG estimations were performed on referral, and at each follow-up visit, using standard radioimmunoassay techniques. Before 1974 urinary hCG estimations were performed. Stage I patients in whom tumour markers were not assayed within 6 weeks of orchidectomy, or where there was over 2 months delay between orchidectomy and radiotherapy, were excluded from the study.

*Staging classification.*—This describes extent of tumour, site(s) of involvement and tumour volume.

I. Lymphogram negative, no evidence of metastases.

II. Lymphogram positive, metastases confined to abdominal nodes. Three sub-groups are recognized:

A. Maximum diameter of metastases < 2 cm
B. Maximum diameter of metastases 2–5 cm
C. Maximum diameter of metastases > 5 cm

III. Involvement of supra- and infradiaphragmatic lymph nodes. No extralymphatic metastases. Abdominal status: A, B, C as for Stage II.

IV. Extralymphatic metastases. Suffixes as follows: $_0$—lymphogram negative, A, B, C as for Stage II. Lung status: $L_1 < 3$ metastases. $L_2$ multiple, $\leq$ 2 cm maximum diameter. $L_3$ multiple, one or more > 2 cm diameter. Liver status: H+ = liver involvement.

Three of the 4 following parameters should be positive before liver involvement is diagnosed.

1. Abnormal liver-function tests
2. Positive CT scan
3. Positive ultrasonic or isotopic scan
4. Clinical enlargement

*Histology.*—This was reviewed in all patients and details of local invasion and cord involvement sought. The histological classification was that proposed by the British Testicular Tumour Panel (BTTP) (Pugh & Cameron, 1976) and included the following sub types:

Malignant teratoma undifferentiated (MTU) [embryonal carcinoma]
Malignant teratoma intermediate (MTI) [teratocarcinoma]
Malignant teratoma trophoblastic (MTT)
Teratoma differentiated (TD)

The presence of a seminoma component did not modify the teratoma classification.

*Pathological staging.*—The local extent of the primary tumour was classified using the criteria of the BTTP (Pugh & Cameron, 1976) as follows:

$P_1$: Tumour confined to testis or rete
$P_2$: Tumour involving epididymis and/or lower cord
$P_3$: Tumour involving upper cord
$P_X$: Staging not possible, inadequate histological material available.

*Immunoperoxidase staining.*—The indirect immunoperoxidase technique was used for tissue staining for hCG (Heyderman & Neville, 1976; Heyderman, 1977). Trophoblastic tissue was used as a positive control, and negative control was achieved by ensuring complete extinction of staining by incubating the anti hCG antisera with hCG before use. Tissue staining for AFP was not assessed, as most tissues had been fixed in formal saline and, in our experience, standard formalin fixation had been associated with a highly variable immunoperoxidase staining pattern for AFP, which we have been unable to interpret with certainty.

*Radiotherapy.*—Stage I patients received radiotherapy (6–8 MeV photons) by anterior and posterior fields to the para-aortic and ipsilateral iliac nodes, delivering mid-plane doses of 40–45 Gray in 4–5 weeks.

*Statistical analysis.*—This was performed according to the techniques described by Peto *et al.* (1976, 1977).

TABLE I.—*Clinical Stage I non-seminomatous germ-cell tumours of the testis (NSGCTT). Relapses according to histology of primary tumour and pathological stage*

| Histology of primary tumour† | Total patients | Pathological stage $P_1$ | $P_2P_3$ | $P_x$ |
|---|---|---|---|---|
| MTU | 14 | 1/7* | 3/5 | 0/2 |
| MTU/SEM | 4 | 1/4 | 0/0 | 0/0 |
| MTI | 26 | 3/20 | 2/2 | 0/4 |
| MTI/SEM | 15 | 2/8 | 1/3 | 1/4 |
| Total | 59 | 7/39 | 6/10 | 1/10 |

$\chi^2 = 7 \cdot 2$
$P < 0 \cdot 01$
* Relapses/total at risk
† MTU = malignant teratoma undifferentiated [embryonal carcinoma]
MTI = malignant teratoma intermediate [teratocarcinoma]
SEM = seminoma

### RESULTS

### (A) *Stage I patients*

*Patient age and side of the tumour.*—The 59 males included in the study ranged in age from 17–60 years (mean 29·6 years) at presentation. The tumour was on the left in 29 patients, right in 29 patients and bilateral in 1 man. Side of presentation and age did not influence outcome of treatment.

*Histology of the primary tumour.*—As shown in Table I, 41/59 patients had MTI and 18/59 MTU. No examples of MMT or TD were seen in this series. The relapse rate for MTU (5/18—27·8%) was slightly but not significantly higher than that for MTI (9/41—21·9%).

*P stage of the primary tumour.*—As shown in Table I, P stage could be assessed in 49 patients. The relapse rate in $P_1$ tumours was 7/39 (17·9%) compared with 6/10 (60%) for $P_2$ and $P_3$ tumours. This difference is significant ($P < 0{\cdot}01$).

*Serum AFP and hCG levels.*—As shown in Table II, initial AFP values (in most cases at referral after orchidectomy) were raised in 18 patients (30·5%). Since there was a mean delay of 20 days between orchidectomy and referral, and since the serum-halving time of AFP is ~7 days,

FIG. 1.—Initial serum α-foetoprotein levels and clinical status after radiotherapy in clinical Stage I non-seminoma germ-cell tumours of the testis (NSGCTT).

TABLE II.—*Serum marker status* at referral in clinical Stage I NSGCTT*

| Raised serum markers | | | | |
|---|---|---|---|---|
| AFP only | AFP (HCG not assayed) | hCG only | AFP & hCG | Both markers negative |
| 13 (22%) | 2 (3%) | 2 (3%) | 3 (5%) | 34 (58%) |

*hCG levels not assayed in 7 patients, 2 of whom had raised AFP levels.

it is possible that the true percentage of AFP-positive patients is higher. Serum AFP levels ranged from 31 to 3000 μg/l, but there was no correlation between serum level and prognosis. Of the 18 patients with raised AFP levels, 5 relapsed (Fig. 1).

Only 5 (8·5%) men had raised hCG levels (Table II), but since the normal half-life of serum hCG is 24–36 h it is highly probable that the true number with raised hCG serum levels at the time of orchidectomy was higher. In 3/5 hCG+ cases, assay was performed on the day of orchidectomy.

*Tissue HCG.*—Tissue sections were stained immunocytochemically for hCG in 48 patients, and were positive in 27 (56·2%). As shown in Table III, in 21 tissue-positive patients, serum hCG levels were normal in 18 when they were referred to the Royal Marsden Hospital. This may well reflect rapid clearance from the blood when the primary tumour is removed. One tissue-negative patient showed high serum levels of hCG. Presence of hCG in tissue sections had no prognostic significance. Six of 27 (22·2%) tissue-positive patients relapsed, compared with 7/21 (33%) tissue-negative patients.

TABLE III.—*Clinical Stage I NSGCTT Serum hCG levels on referral in relation to tissue hCG*

| Immunocytochemically demonstrated tissue hCG | Serum hCG | | |
|---|---|---|---|
| | High | Low | ND |
| + | 3 | 21 | 3 |
| − | 1* | 16 | 4 |
| ND | 1 | 10 | 0 |

* Possibly a sampling error: few sections available for analysis.

*Changes in serum marker levels between orchidectomy and radiotherapy.*—As shown in Table IV, patients were divided into two groups, A and B.

In *Group A* there was evidence that serum marker levels were either persistently negative before irradiation or, if high, they fell with a half-life consistent with the removal of all the tumour (AFP: 6–7 days, hCG: 24–36 h).

*Group B* included patients with persistently high serum marker levels, and those with a slow rate of fall after orchidectomy. If only one value was available several weeks after orchidectomy and before

TABLE IV.—*Clinical Stage I. Behaviour of serum markers after orchidectomy in relation to relapse rate*

| Group | Criteria | Number of patients | No. of relapsing (%) |
|---|---|---|---|
| A | Markers always negative, or high marker at orchidectomy falling with normal half-life AFP 7 days hCG < 36 h | 49 | 8 (16) |
| B | High marker level with a) rate of fall protracted or b) data not permitting rate of marker fall to be determined, but inconsistent with rapid clearance | 10 | 6 (60) |

A *vs* B = $P < 0{\cdot}01$.

FIG. 2.—Clinical Stage I NSGCTT. Continuous disease-free survival by serum-marker status. Group A = serum markers persistently negative or if high before radiotherapy fell rapidly after orchidectomy. Group B = serum markers showing protracted fall or persistent elevation below orchidectomy and irradiation. (The Royal Marsden Hospital 1973–1978).

radiotherapy, it was assumed that complete clearance following removal of the primary tumour would not occur. Of the total group, 6 patients had 2–6 measurements and 4 patients had one.

In *Group A*, 8/49 (16·3%) patients relapsed, compared with 6/10 (60%) of

FIG. 3.—Histology NSGCTT of the primary tumour in relation to absence (Stage I) or presence (Stage IV) of pulmonary metastases in patients with no clinical evidence of lymph-node metastases.

FIG. 4.—Histology NSGCTT of primary tumour in relation to absence (Stage I) or presence (Stage IV) of pulmonary metastases in patients with no clinical evidence of lymph-node metastases.

Group B patients. This difference is significant ($P < 0{\cdot}01$). The disease-free survival curves are shown in Fig. 2.

*Relapse pattern and patient survival.*—Of the 14 patients who relapsed, the pattern was as follows: left cervical nodes (3), lung ± mediastinal nodes (7), brain/lung (1), groin nodes (1), abdominal nodes + extralymphatic metastases (2). Seven patients were successfully treated for relapse and 52/59 (88%) of the patients are alive and disease-free. There were 5 (8%) tumour deaths, 1 died of a cerebrovascular accident and 1 of complications relating to chemotherapy.

(B) *Stage* $IV_0$ $L_1$ $L_2$ *and* $L_3$ *patients*

This group was examined with respect to histology and pathological stage of the primary tumour, and compared with the findings described above in Stage I disease.

Fig. 3 shows that, whereas in Stage I there is a preponderance of MTI primary tumours, the commonest sub-type in $IV_0$ $L_{1-3}$ is MTU.

As shown in Fig. 4, whereas there was a preponderance of $P_1$ tumours in Stage

I ($30/49 = 79 \cdot 6\%$) the converse was the case in $IV_0$ $L_{1-3}$, where 9/11 (82%) of men in whom adequate material was available for P stage to be established showed evidence of cord invasion ($P_2P_3$).

If P stage and histology for Stage I and $IV_0$ $L_{1-3}$ patients are considered together (Fig. 5) it is seen that MTI tends to be associated with $P_1$ primary extent (28/35—80%), whereas MTU shows a higher tendency to involve the spermatic cord (11/24—46%).

### DISCUSSION

In an attempt to understand factors influencing treatment outcome we have studied 59 patients with clinical Stage I NSGCTT managed by orchidectomy and radiotherapy. Of these, 14 patients (24%) relapsed and 45 (76%) have remained persistently disease-free.

Age at presentation and side of primary tumour had no influence on relapse rate. In previous analyses on a larger number of patients the relapse rate for patients with MTU primaries was significantly greater than that for MTI (Peckham *et al.*, 1977). In the present smaller series no significant difference was observed. The observations made in Stage I patients and patients presenting with lung metastases and no evidence of lymph-node disease indicate that MTU is associated with a higher probability of cord involvement than is MTI.

Extent of the primary tumour was a significant prognostic factor, there being an increased incidence of relapses in patients with tumour involving spermatic cord, epididymis or scrotal sac ($P < 0 \cdot 01$). This agrees well with the increased incidence of $P_2$ and $P_3$ stages in a group of patients presumed to have haematogenous spread without clinical evidence of nodal deposits at presentation. An important prognostic indicator in this series was the rate of decline of serum AFP and hCG levels after orchidectomy. Thus the relapse rate in patients with negative or rapidly falling markers was significantly less than in patients with a protracted decline (16% *vs* 60% respectively; $P < 0 \cdot 01$).

Because of the delay in referral following orchidectomy, the estimated incidences of 30·5% for patients with high AFP levels is probably artificially low, since the half-life of serum AFP is $\sim 7$ days. Since the average delay in referral was 21 days, patients with pre-orchidectomy levels of 25–200 $\mu$g/l may have had undetectable levels on referral. This applies even more to hCG, the half-life of which is 24–36 h. This may well explain why only 3/27 patients with immunocytochemically demonstrable hCG in primary tumour tissue had high serum hCG levels on referral. The frequency of hCG+ tissue (56·2%) may reflect the proportion of patients with elevated serum hCG levels more accurately. The presence of immunocytochemically demonstrable hCG did not influence prognosis. Similarly, no significant difference was observed in rates of relapse between patients with initially normal and high serum marker levels (Fig. 1). However, because of the delay in most patients between orchidectomy and first assay, and the possibility that a proportion of men with normal serum marker levels at referral may have had high levels at orchidectomy, the true significance of serum-marker status cannot be fully assessed.

The present study has the disadvantage of being a retrospective analysis of a relatively small number of patients who were eligible for inclusion. Nevertheless, it seems clear that extent of primary tumour and serum marker behaviour after orchidectomy are important factors which discriminate between patients with low and high risk of extralymphatic dissemination. In Stage I patients with persistent serum markers there is a high risk of extralymphatic disease, and the treatment of choice is chemotherapy. In patients free of adverse prognostic factors, the relapse rate after lymph-node irradiation is low, and the overall cure rate high if relapses are detected early and treated promptly with chemotherapy (Pechkam *et al.*, 1979, 1981). On the other hand, it seems probable that at least 75% of men with Stage I disease and no adverse prognostic factors may be cured by orchidectomy alone. In 1979 radiotherapy was abandoned in this group of patients in favour of a policy of careful follow-up after orchidectomy. To date, of 21 men followed up for at least a year, 4 (19%) have relapsed. All relapses have occurred within 6 months of orchidectomy, and have only been seen in patients with MTU (embryonal carcinoma). All 4 relapsed patients have been successfully treated with chemotherapy for small-volume disease. The objective of the surveillance study is to define prognostic factors accurately, in order to provide a rational basis for future management. In the retrospective analysis which is described in this report we were unable with confidence to distinguish $P_2$ from $P_3$ tumours, because of limited pathological material. For this reason, unless there is tumour in the proximal spermatic cord, patients are not excluded from the surveillance study. Other potentially important factors, such as lymphatic permeation and vascular invasion within the primary tumour, are being investigated in conjunction with a more detailed study of blood and tissue markers.

The authors wish to thank Professor K. D. Bagshawe and Miss A. H. Orr for assaying hCG and AFP levels.

### REFERENCES

HEYDERMAN, E. & NEVILLE, A. M. (1976) Syncytiotrophoblasts in malignant testicular tumours *Lancet*, ii, 103.

HEYDERMAN, E. (1977) Immunoperoxide technique in histopathology: Application, methods and controls. *J. Clin. Pathol.*, **32**, 971.

HUSBAND, J. E., BARRETT, A. & PECKHAM, M. J. (1981) Evaluation of computed tomography in the management of testicular teratoma *Br. J. Urol.*, **53**, 179.

PECKHAM, M. J. (1979) An appraisal of the role of radiation therapy in the management of non seminomatous germ-cell tumors of the testis in the era of effective chemotherapy *Cancer Treat. Rep.*, **63**, 1653.

PECKHAM, M. J., BARRETT, A., MCELWAIN, T. J. & HENDRY, W. F. (1979) Combined management of malignant teratoma of the testis *Lancet*, ii, 267.

PECKHAM, M. J., BARRETT, A., MCELWAIN, T. J., HENDRY, W. F. & RAGHAVAN, D. (1981) Non-seminoma germ cell tumours (malignant teratoma) of the testis: Results of treatment and an analysis of prognostic factors *Br. J. Urol.*, **53**, 162.

PECKHAM, M. J., HENDRY, W. F., MCELWAIN, T. J. & CALMAN, F. M. B. (1977) The multimodality management of testicular teratomas. In *Adjuvant Therapy of Cancer* (Ed. Salmon & Jones). Amsterdam: North-Holland Publishing Company, p. 305.

PETO, R., PIKE, M. C., ARMITAGE, P. & 7 others (1976 & 1977) Design and analysis of randomised clinical trials requiring prolonged observation of each patient. Parts I & II *Br. J. Cancer*, **34**, 585; **35**, 1.

PUGH, R. C. B. & CAMERON, K. M. (1976) Teratoma. In *Pathology of the Testis*, (Ed. Pugh) London: Blackwell. p. 199.